# Supplementary figures and images for: Diverse laboratory colonies of Aedes aegypti harbor the same adult midgut bacterial microbiome
Source: Parasit Vectors. 2018 Mar 27;11:207. doi: 10.1186/s13071-018-2780-1 (PMC5870067; doi:10.1186/s13071-018-2780-1)

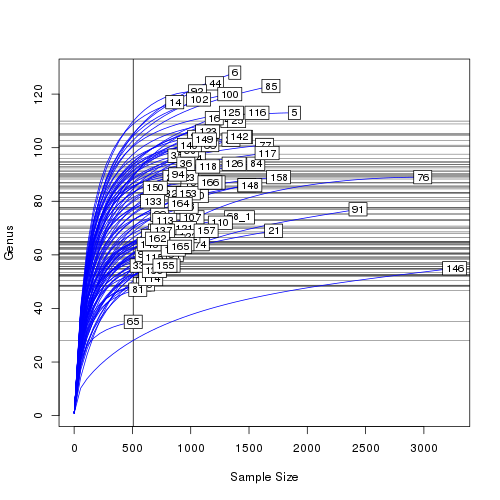

Supplement: Supplementary file 1 — Figure S1. Rarefaction curves for the individual samples used in the analysis at the genus level. The curves show the number of detected bacterial genera as a function of the number of reads analyzed per sequencing library. Each curve represents a single midgut sample. (PNG 58 kb) [file 13071_2018_2780_MOESM1_ESM.png]

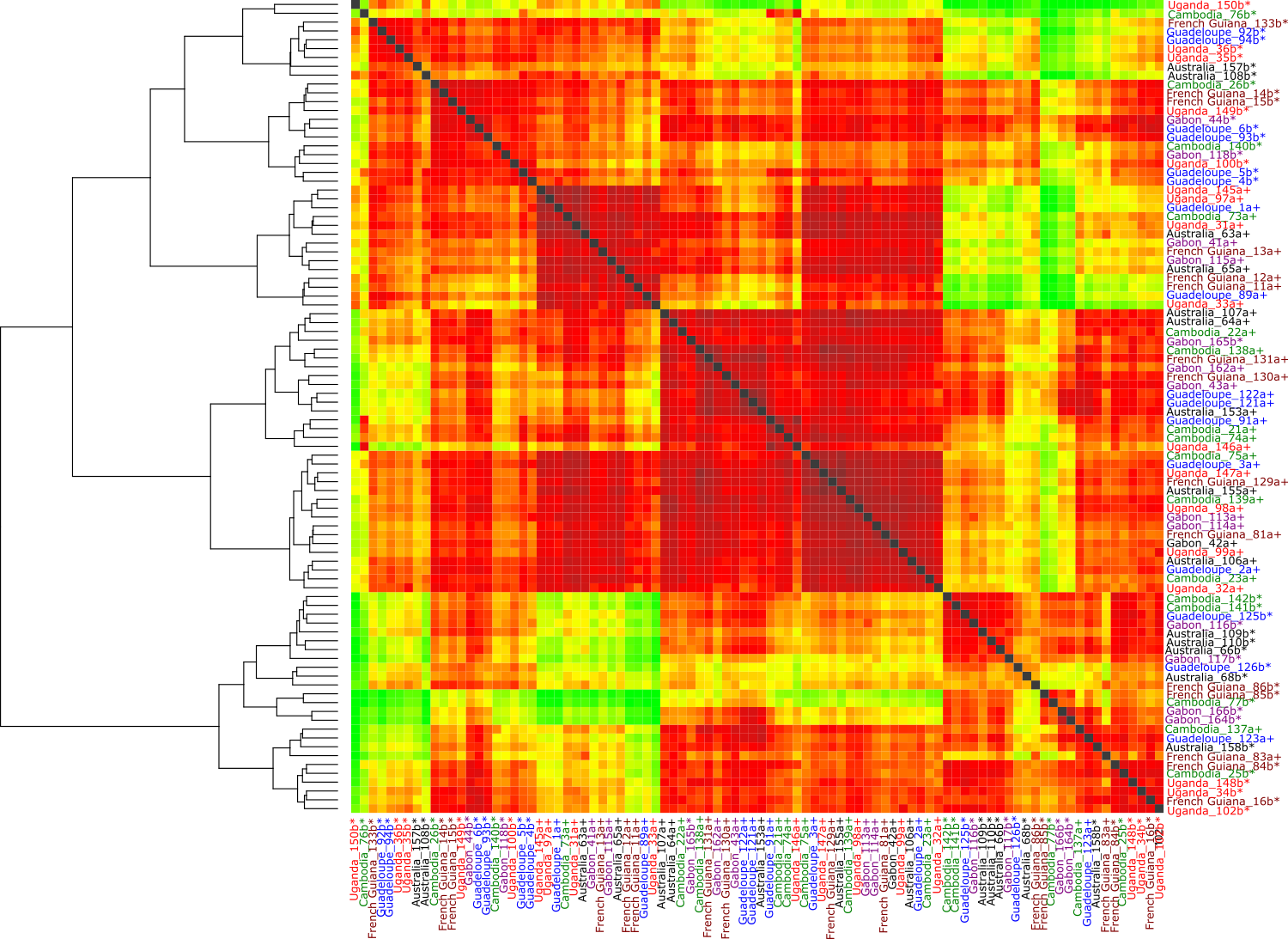

Supplement: Supplementary file 3 — Figure S3. OTU-independent analysis of midgut bacterial community structure confirms the lack of clustering by colony. The heat map of Bray-Curtis dissimilarity index based on k-mer abundance and hierarchical clustering shows that the sequences are structured based on the sequencing run and the day of DNA extraction, not the laboratory colony of origin. Sample names are color coded by colony and labeled according to the DNA extraction batch (a and b) and the sequencing run (* and +). In the heat map, red color indicates high similarity, whereas green color indicates low similarity. (PNG 476 kb) [file 13071_2018_2780_MOESM3_ESM.png]
